# The association between immune-related adverse events and survival outcomes in Asian patients with advanced melanoma receiving anti-PD-1 antibodies

**DOI:** 10.1186/s12885-020-07508-7

**Published:** 2020-10-21

**Authors:** Chiao-En Wu, Chan-Keng Yang, Meng-Ting Peng, Pei-Wei Huang, Ching-Fu Chang, Kun-Yun Yeh, Chun-Bing Chen, Chih-Liang Wang, Chao-Wei Hsu, I-Wen Chen, Cheng-Tao Lin, Shir-Hwa Ueng, Gigin Lin, Yu-Fen Lin, Chi-Yuan Cheng, John Wen-Cheng Chang

**Affiliations:** 1 Division of Haematology-Oncology, Department of Internal Medicine, Chang Gung Memorial Hospital, Linkou, Taiwan; 2 Chang Gung University College of Medicine, 5, Fu-Hsing Street, Kwei-Shan, Taoyuan, Taiwan; 3 Immuno-Oncology Center of Excellence, Chang Gung Memorial Hospital at Linkou, Taoyuan, Taiwan; 4 Division of Hemato-Oncology, Department of Internal Medicine, Chang Gung Memorial Hospital, Keelung, Taiwan; 5 Chang Gung University College of Medicine, Taoyuan, Taiwan; 6 Department of Dermatology, Chang Gung Memorial Hospital at Linkou, Taipei, Keelung Taiwan; 7 Department of Thoracic Medicine, Chang Gung Memorial Hospital, Linkou, Taiwan; 8 Department of Gastroenterology and Hepatology, Chang Gung Memorial Hospital, Linkou, Taiwan; 9 Division of Endocrinology and Metabolism, Department of Internal Medicine, Chang Gung Memorial Hospital, Linkou, Taiwan; 10 Department of Obstetrics and Gynecology, , Chang Gung Memorial Hospital, Linkou, Taiwan; 11 Department of Pathology, Chang Gung Memorial Hospital, Linkou, Taiwan; 12 Department of Medical Imaging & Intervention, Chang Gung Memorial Hospital, Linkou, Taiwan; 13 Department of Nursing, Chang Gung Memorial Hospital, Linkou, Taiwan; 14 Department of Pharmacy, , Chang Gung Memorial Hospital, Linkou, Taiwan

**Keywords:** Immune checkpoint inhibitors, irAE, Skin toxicity, Vitiligo, Endocrine, Melanoma, PD-1

## Abstract

**Background:**

The association between immune-related adverse events (irAEs) and survival outcomes in patients with advanced melanoma receiving therapy with immune checkpoint inhibitors (ICIs) has not been well established, particularly in Asian melanoma.

**Methods:**

We retrospectively reviewed 49 melanoma patients undergoing therapy with ICIs (anti-PD-1 monotherapy), and analyzed the correlation between irAEs and clinical outcomes including progression-free survival (PFS) and overall survival (OS). Results: Overall, the patients who experienced grade 1–2 irAEs had longer PFS (median PFS, 4.6 vs. 2.5 months; HR, 0.52; 95% CI: 0.27–0.98; *p* = 0.042) and OS (median OS, 15.2 vs. 5.7 months; HR, 0.50; 95% CI: 0.24–1.02; *p* = 0.058) than the patients who did not experience irAEs. Regarding the type of irAE, the patients with either skin/vitiligo or endocrine irAEs showed better PFS (median PFS, 6.1 vs. 2.7 months; HR, 0.40, 95% CI: 0.21–0.74; *p* = 0.003) and OS (median OS, 18.7 vs. 4.5 months; HR, 0.34, 95% CI: 0.17–0.69, p = 0.003) than patients without any of these irAEs.

**Conclusions:**

Melanoma patients undergoing anti-PD-1 monotherapy and experiencing mild-to-moderate irAEs (grade 1–2), particularly skin (vitiligo)/endocrine irAEs had favorable survival outcomes. Therefore, the association between irAEs and the clinical outcomes in melanoma patients undergoing anti-PD-1 ICIs may be severity and type dependent.

## Background

Immune checkpoint inhibitors (ICIs), including ipilimumab, nivolumab, and pembrolizumab, have become standard therapies in advanced melanoma, regardless of genetic alteration [1–3]. The combination of ipilimumab and nivolumab demonstrated greater effectiveness than monotherapy with either of these [4–7]; however, combination therapy had the greater toxicity than monotherapy and insufficient power for overall survival (OS) over nivolumab limit the application of combination treatment in advanced melanoma. ICIs induce favorable response in some patients, so biomarkers are needed to determine the clinical course of ICIs in advanced melanoma.

One potentially effective clinical biomarker of ICI response in cancer patients is immune-related adverse event (irAE) [8]. Patients who experience irAEs during therapy with anti-PD-1 and anti-PD-L1 antibodies have been found to show favorable outcomes – overall response rate (ORR), progression-free survival (PFS), and OS – in cases of melanoma, lung cancer, and urothelial carcinoma [9–15]. However, as compared with other cancer types, the association between irAE occurrence and anti-PD-1 antibody efficacy is not well established in metastatic melanoma patients. Several retrospective studies have reported improved outcomes in patients who experience irAEs; however, not all measured outcomes consistently improved in these patients [16–19]. Besides, given most of the previous publications are studied on cutaneous melanoma, it raises the question that whether those results could be applied in Asian in which acral and mucosal melanoma are predominant.

Previously, we reported the clinical efficacy and safety of ICIs in our institution in 80 ICI-naïve melanoma patients [20]. As anti-CTLA-4, anti-PD-1 antibodies, and their combination showed distinct irAE patterns [2], we selected the patients undergoing anti-PD-l monotherapy, with either nivolumab or pembrolizumab, and analyzed the association between irAEs and survival outcomes. This study could provide additional evidence of irAEs as biomarkers of treatment outcomes.

## Methods

### Patients

All patients with histologically confirmed melanoma treated at the Chang Gung Memorial Hospital (CGMH), Linkou, during 2014 to 2019, were retrospectively reviewed. ICI-naïve patients undergoing anti-PD-1 antibody treatment were included in the current study. Only unresectable stage III and IV melanomas were included in current study. The patients with stage IV melanomas undergoing complete resection were excluded as ICI was used for adjuvant treatment in such cases. Patients who received other systemic treatments prior to ICI therapy, such as chemotherapy, targeted therapy, or cytokine therapy, were also included. A total of 49 ICI-naïve patients with advanced melanoma receiving anti-PD-1 antibodies, either nivolumab or pembrolizumab, were included in the study. The last follow-up timepoint included in the study was March 31, 2020.

### Treatment regimens and response evaluation

The patients were treated with anti-PD-1 antibodies, either nivolumab (3 mg/kg every 2 weeks) or pembrolizumab (2 mg/kg every 3 weeks), until disease progression or intolerable toxicity. The dose or schedule of anti-PD-1 ICIs was adjusted by the physicians based on the patients’ clinical condition and toxicity from treatment. Laboratory data on liver, renal, and endocrine function; cardiac enzymes; viral hepatitis status; and autoimmune antibodies were obtained before treatment and followed up regularly after starting treatment. Tumor response was evaluated by regular physical examination, chest radiography, computed tomography, or positron emission tomography.

### Patient characteristics and evaluation of outcomes

Patient characteristics, including age, sex, Eastern Cooperative Oncology Group (ECOG) performance status, systemic treatment prior to ICIs, stage of melanoma, histologic types, and location of primary melanoma were recorded.

The irAEs were evaluated by a clinician based on the findings of laboratory tests, clinical examinations, and imaging studies. Cases with suspicious irAEs were discussed at meetings held by the Immuno-Oncology Center of Excellence of Chang Gung Memorial Hospital at Linkou, consisting of medical oncologists, pulmonologists, hepatologists, endoscopists, endocrinologists, dermatologists, neurologists, radiologists, nurses, etc. The irAEs (with a potential immunologic cause) were graded according to the National Cancer Institute Common Terminology Criteria for Adverse Events, version 4.0.

The principle of irAE management follows the clinical guideline and may be adjusted based on physicians’ judgement and team discussion [21]. Generally, ICIs were withheld temporarily in the patients with mild-moderate irAE (grade 1–2) and systemic glucocorticoids / suppressants were not needed. Rechallenge of ICIs was applied when patients recovered from irAE. In contrast, systemic glucocorticoids/suppressants were applied in patients grade 3 and more irAE.

The RECIST (Response Evaluation Criteria in Solid Tumors) 1.1 Criteria were used to evaluate the best tumor response. PFS was defined as the length of time from the first day of ICI treatment until the first clinical or radiological evidence of disease progression, death, or latest follow-up timepoint. OS was defined as the length of time from the first day of ICI treatment until the date of death or last follow-up.

### Statistical analysis

The PFS and OS were estimated by the Kaplan-Meier method and compared by the log-rank test. Univariate analysis was performed to evaluate possible prognostic factors, and the results were presented as hazard radio (HR) and confidence interval (CI). IBM SPSS Statistics for Windows (Version 20.0, Armonk, NY, USA) was used for statistical analyses, where *P* < 0.05 was considered statistically significant. This study was approved by the Institutional Review Board of CGMH (202000182B0). Patient consent to participate was not required because of the retrospective nature of this study, which was approved by the Institutional Review Board of CGMH.

## Results

### Patient characteristics

In the current study, a total of 49 advanced melanoma patients undergoing anti-PD-1 monotherapy (33 with nivolumab and 16 with pembrolizumab) as their first-line ICI treatment were included for retrospective analysis. Eighteen (36.7%) received prior systemic treatments such as targeted therapy, cytokine therapy, or chemotherapy. The median age was 61 years. Twenty-two (44.9%) were male and 27 (55.1%) were female. Forty-two (85.7%) patients had good performance status of 0–1. Fifteen patients (30.6%) had acral melanoma, 16 (32.7%) had mucosal melanoma, and only five (10.2%) had nonacral cutaneous melanoma. Six patients had locally advanced melanoma (stage III, 12.2%), and 43 had metastatic melanoma (stage IV, 87.8%). The patients’ characteristics are summarized in Table 1. The median PFS and median OS were 3.1 (95% CI: 2.8–3.5) and 10.7 (95% CI: 7.7–13.8) months, respectively (Supplementary Fig. S1). The ORR was 10.2% (*n* = 5) including 2 (4.1%) complete response.

**Table 1:** Patients’ Characteristics

Characteristics	Number	%
Age (years)		
Median (IQR)	61 (18)	
≦60	24	49.0
> 60	25	51.0
Gender		
Male	22	44.9
Female	27	55.1
Performance status		
0	29	59.2
1	13	26.5
2	6	12.2
3	1	2.0
Location of primary site		
Extremities	18	36.7
Head and neck	9	18.4
Trunk	16	32.7
Unknown	6	12.2
Type		
Acral melanoma	15	30.6
Non-acral cutaneous melanoma	5	10.2
Mucosal melanoma	16	32.7
Others	7	14.3
Unknown	6	12.2
Stage		
3	6	12.2
4, M1a	5	10.2
4, M1b	8	16.3
4, M1c	30	61.2
BRAF gene mutation		
No	34	69.4
Yes	9	18.4
Unknown	6	12.2
Treatment regimen		
Nivolumab	33	67.3
Pembrolizumab	16	32.7
Immunotherapy therapy		
First-line	31	63.3
Second-or later-line	18	36.7
Response		
CR	2	4.1
PR	3	6.1
SD	9	18.4
PD	29	59.2
N/A	6	12.2

*IQR* interquartile range, *CR* complete response, *PR* partial response, *SD* stable disease, *PD* progressive disease, *N/A* not assessed

### The pattern of irAE occurrence

**Fig. 1:**
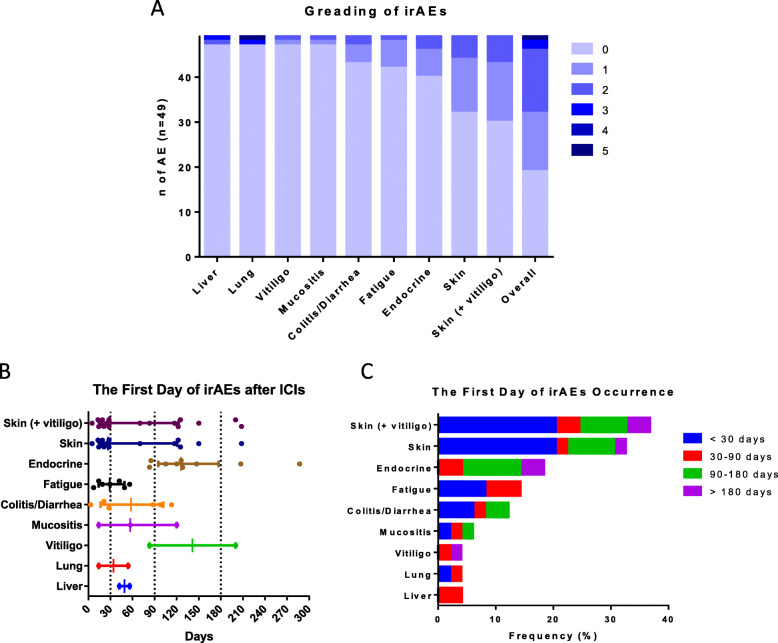
Severity and types of irAEs in melanoma patients receiving anti-PD-1 antibodies. **a** The numbers of patients experiencing different grades and types of irAEs. **b** The first day of irAEs after ICIs based on different types of irAEs. **e** Grouping based on the first day of irAE occurrence. irAEs, immune-related adverse events; ICIs, immune checkpoint inhibitors

The median follow-up time was 9.1 months (range: 0.3–48.3 months). Overall, 30 (61.2%) patients experienced irAEs of various etiologies. Three patients experienced severe irAE (grade 3–5) and one died of irAE of pneumonitis (grade 5). Another two patients had grade 3 pneumonitis and grade 3 hepatitis respectively. Skin lesions (*n* = 19, 38.8%) was the most common irAE followed by endocrine irAE (*n* = 9, 19.4%), fatigue (*n* = 7, 14.3%), and colitis/diarrhea (*n* = 6, 12.2%). The frequency of mucositis, vitiligo, liver- and lung-related irAEs were relatively low (< 5%) (Fig. 1a and Supplementary Table 1). In terms of vitiligo, some previous studies reported it is an irAE of interest in melanoma patients treated with ICIs and a possible predictor of favorable response and survival. However, only two patients had vitiligo in the current study so it was merged with other skin AEs for analyses (Figs. 1, 3, and Tables 2, 3).

**Table 2:** Univariate analysis of prognostic factors in progression-free survival

Adverse events	Median (months)	95% C.I. of median	*P* value	Hazard ratio	95% C.I. of HR	P value
Skin			.274			
Grade 0 (*n* = 32)	2.8	1.9–3.8		1		
Grade 1–2 (*n* = 17)	3.6	1.6–5.6		0.71	0.38–1.32	.278
Skin/vitiligo			.092			
Grade 0 (*n* = 30)	2.5	1.6–3.4		1		
Grade 1–2 (n = 19)	4.6	2.5–6.7		0.59	0.32–1.10	.096
Mucositis			.287			
Grade 0 (*n* = 46)	3.1	2.5–3.8		1		
Grade 1–2 (n = 3)	3.0	0.4–5.6		1.90	0.57–6.29	.296
Colitis			.624			
Grade 0 (*n* = 43)	3.2	2.6–3.7		1		
Grade 1–2 (n = 6)	3.0	2.3–3.7		1.24	0.52–2.96	.626
Liver			.353			
Grade 0 (*n* = 47)	3.2	2.7–3.6		1		
Grade 1–2 (n = 1)	3.1	–		1.48	0.20–10.99	.700
Grade 3–5 (n = 1)	2.1	–		3.89	0.50–30.19	.194
Lung			.005			
Grade 0 (n = 47)	3.2	2.8–3.5		1		
Grade 3–5 (n = 2)	0.9	–		6.71	1.44–31.38	.016
Endocrine			.094			
Grade 0 (*n* = 40)	2.7	2.1–3.3		1		
Grade 1–2 (n = 9)	6.1	2.7–9.6		0.52	0.24–1.13	.100
Fatigue			.024			
Grade 0 (*n* = 42)	3.2	2.7–3.7		1		
Grade 1–2 (n = 7)	2.4	1.5–3.4		2.56	1.09–6.00	.030
Vitiligo			.234			
Grade 0 (n = 47)	3.0	2.5–3.4		1		
Grade 1–2 (n = 2)	4.7	–		0.32	0.04–2.33	.261
Skin/vitiligo/endocrine			.003			
Grade 0 (*n* = 26)	2.3	1.7–2.8		1		
Any grade (*n* = 23)	4.8	4.3–5.3		0.40	0.21–0.74	.003
Overall			.001			
Grade 0 (n = 19)	2.5	1.7–3.3		1		
Grade 1–2 (*n* = 27)	4.6	2.8–6.4		0.52	0.27–0.98	.042
Grade 3–5 (n = 3)	2.0	0.2–3.8		4.24	1.1–16.3	.035

*C.I.* confidence interval

**Table 3:** Univariate analysis of prognostic factors in overall survival

Adverse events	Median (months)	95% C.I. of median	P value	Hazard ratio	95% C.I. of HR	P value
Skin			.054			
Grade 0 (*n* = 32)	5.7	1.3–10.0		1		
Grade 1–2 (n = 17)	16.4	4.8–28.0		0.49	0.24–1.03	.059
Skin/vitiligo			.047			
Grade 0 (n = 30)	5.1	3.1–7.2		1		
Grade 1–2 (n = 19)	16.4	4.8–28.0		0.49	0.24–1.01	.052
Mucositis			.150			
Grade 0 (n = 46)	11.3	5.6–17.0		1		
Grade 1–2 (n = 3)	6.0	0.1–13.2		2.36	0.71–7.92	.163
Colitis			.773			
Grade 0 (n = 43)	13.1	6.9–19.4		1		
Grade 1–2 (n = 6)	9.7	2.716.6		1.15	0.44–2.98	.773
Liver			.292			
Grade 0 (n = 47)	10.7	5.3–16.2		1		
Grade 1–2 (n = 1)	9.7	–		1.55	0.21–11.50	.670
Grade 3–5 (n = 1)	2.8	–		4.33	0.55–33.91	.162
Lung			.001			
Grade 0 (n = 47)	10.7	6.2–15.3		1		
Grade 3–5 (n = 2)	0.9	–		8.90	1.83–43.4	.007
Endocrine			.062			
Grade 0 (n = 40)	8.8	2.4–15.3		1		
Grade 1–2 (n = 9)	18.7	2.0–35.3		0.38	0.13–1.09	.073
Fatigue			.019			
Grade 0 (n = 42)	11.3	6.1–16.4		1		
Grade 1–2 (n = 7)	3.7	1.3–6.2		2.69	1.14–6.34	.024
Vitiligo			.856			
Grade 0 (n = 47)	10.7	7.3–14.2		1		
Grade 1–2 (n = 2)	8.1	–		0.83	0.11–6.16	.856
Skin/vitiligo/endocrine			.002			
Grade 0 (n = 26)	4.5	0.9–8.1		1		
Any grade (n = 23)	18.7	6.2–31.1		0.34	0.17–0.69	.003
Overall			<.001			
Grade 0 (n = 19)	5.7	0.1–11.7		1		
Grade 1–2 (n = 27)	15.2	7.9–22.5		0.50	0.24–1.02	.058
Grade 3–5 (n = 3)	2.0	0.2–3.8		5.13	1.29–20.34	.020

*C.I*. confidence interval

Regarding the onset of irAEs, most skin-related irAEs occurred within the first month, but a few patients (*n* = 2) experienced skin-related irAEs after 6 months of exposure to anti-PD-l antibodies. All endocrine-related irAEs occurred after 80-day exposure to anti-PD-l antibodies. Fatigue occurred within the first 2 months. Two distinct patterns were found for diarrhea/colitis as half of the instances occurred within 1 month and the other half, after 3 months (Fig. 1b-c).

### The association between irAE and PFS

**Fig. 2:**
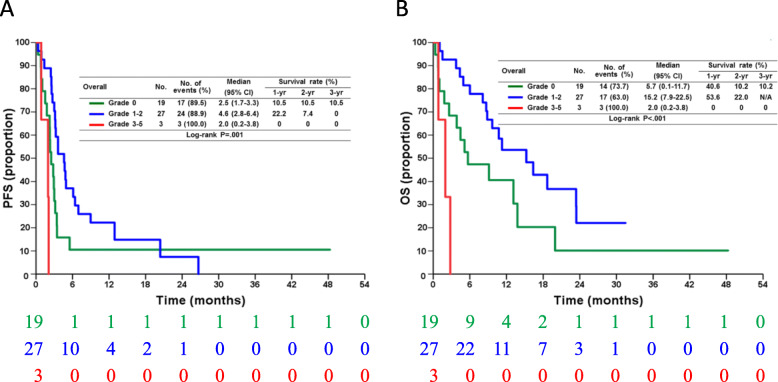
Progression-free survival (PFS) (**a**) and overall survival (OS) (**b**) in melanoma patients undergoing anti-PD-1 antibodies based on severity of irAE The patients who experienced no irAEs, mild irAEs (grade 1–2), and severe irAEs (grade 3–5) had significantly different PFS (*p* = 0.001) and OS (*p* < 0.001). The patients with mild irAEs showed favorable survival and those with severe irAEs showed unfavorable survival. The numbers below the charts correspond to patients at risk at each time point. irAEs, immune-related adverse events

Univariant analysis was performed to analyze the association between irAE and survival outcomes. Regarding overall irAEs, the patients who experienced grade 1–2 irAEs had significantly longer PFS than those who did not experience irAEs (median PFS, 4.6 vs. 2.5 months; HR, 0.52; 95% CI: 0.27–0.98; *p* = 0.042). In contrast, the patients who experienced severe irAEs (grade 3–5) had shorter PFS than those who did not experience any irAE (median PFS, 2.0 vs. 2.5 months; HR, 4.24; 95% CI: 1.1–16.3; *p* = 0.035) (Fig. 2a, Table 2).

**Fig. 3:**
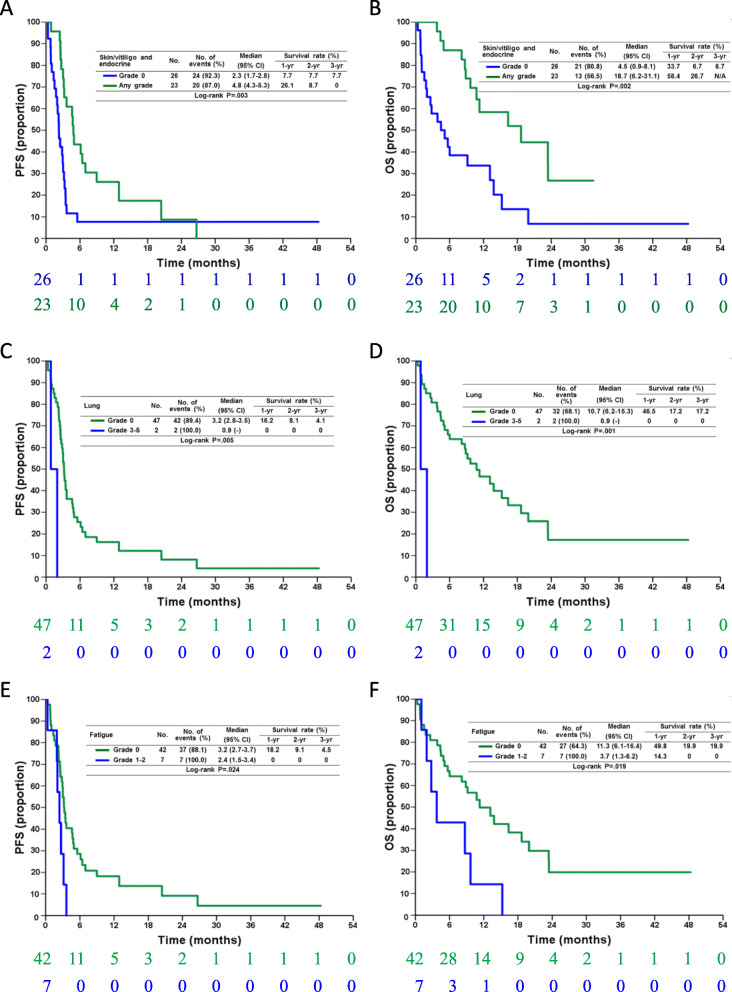
Progression-free survival (PFS) (**a**, **c**, **e**) and overall survival (OS) (**b**, **d**, **f**) in melanoma patients receiving anti-PD-1 antibodies, who had skin/vitiligo/endocrine (**a**, **b**), and lung (**c**, **d**) irAEs and fatigue (**e**, **f**). The patients experiencing skin/vitiligo/endocrine irAEs had favorable PFS and OS but those who experienced fatigue and lung irAEs had unfavorable PFS and OS. The numbers below the charts correspond to patients at risk at each time point. irAE, immune-related adverse events

Further, the patients with grade 1–2 skin/vitiligo (median PFS, 4.6 vs. 2.5 months; HR, 0.59; 95% CI: 0.32–1.10; *p* = 0.096) or grade 1–2 endocrine irAEs (median PFS, 6.1 vs. 2.7 months; HR, 0.52; 95% CI: 0.24–1.13; *p* = 0.100) showed favorable PFS without statistically significance (Supplementary Fig. S2). The patients with either irAEs had significantly better PFS than patients without skin/vitiligo or endocrine irAEs (median PFS, 6.1 vs. 2.7 months; HR, 0.40; 95% CI: 0.21–0.74, *p* = 0.003) (Fig. 3a).

Unlike skin/vitiligo and endocrine irAEs, the patients with grade 1–2 fatigue (median PFS, 2.4 vs. 3.2 months; HR, 2.56; 95% CI: 1.09–6.00; *p* = 0.030) had significantly worse PFS than the patients without fatigue (Fig. 3c). In addition, the patients with lung-related irAEs had significantly worse PFS (median PFS, 0.9 vs. 3.2 months; HR, 6.71, 95% CI: 1.44–31.38; *p* = 0.016) than the patients without those irAEs (Fig. 3e). Of note, only two patients experienced lung-related irAEs and both had grade 3–5 irAEs, so the influence of grade 1–2 lung-related irAEs is unknown in the current study.

### The association between irAEs and OS

Regarding the association between irAEs and OS, the patients who experienced grade 1–2 irAEs showed longer OS than the patients without irAEs (median OS, 15.2 vs. 5.7 months; HR, 0.50; 95% CI: 0.24–1.02; *p* = 0.058). In contrast, the patents who experienced severe irAEs (grade 3–5) had shorter OS than the patients without any irAE (median OS, 2.0 vs. 5.7 months; HR, 5.13; 95% CI: 1.29–20.34; *p* = 0.020) (Fig. 2b, Table 3). For different types of irAEs, the patients with grade 1–2 skin/vitiligo (median OS, 16.4 vs. 5.1 months; HR, 0.49; 95% CI: 0.24–1.01; *p* = 0.052) or grade 1–2 endocrine irAEs (median OS, 18.7 vs. 8.8 months; HR, 0.38; 95% CI: 0.24–1.13; *p* = 0.073) showed favorable OS (Supplementary Fig. S2). On combining skin/vitiligo and endocrine irAEs, we found that the patients with either irAE had significantly better PFS than patients without these irAEs (median OS, 18.7 vs. 4.5 months; HR, 0.34; 95% CI: 0.17–0.69, *p* = 0.003) (Fig. 3a).

Similar to the impact of fatigue in PFS, the patients with grade 1–2 fatigue (median OS, 3.7 vs. 11.3 months; HR, 2.69; 95% CI: 1.14–6.34; *p* = 0.024) had significantly shorter OS than the patients without fatigue (Fig. 3d). In addition, the patients with lung-related irAEs had significantly shorter OS (median OS, 0.9 vs. 10.7 months; HR, 8.90; 95% CI: 1.83–43.4; *p* = 0.007) than the patients without lung-related irAEs (Fig. 3e).

## Discussion

In the current study, the association between irAEs and clinical outcomes was investigated using data from 49 patients treated with anti-PD-1 antibodies in single institute cancer center; it was found that the association was dependent on type and severity of the irAEs. The patients with mild-to-moderate irAEs (grade 1–2) had better PFS and OS. In addition, patients with skin irAEs/vitiligo/endocrine irAEs showed favorable PFS and OS.

Our findings were compatible with recently published meta-analysis investigating irAE and efficacy of ICIs and showing the occurrence of irAEs was significantly associated with a better ICI efficacy in cancer patients, particularly endocrine, skin, and low-grade irAEs [22]. In addition, this association was limited in PD-1 monotherapy but not anti-CTLA-4 nor combination therapy indicating different targets of ICIs might be associated with distinct irAE patterns which should be analyzed and discussed separately. Furthermore, ethnicity and melanoma subtype was found to be associated with distinct irAE profiling in the setting of PD-1 ICI by using 3 independent melanoma centers from the US. and China [23]. This finding indicates that not only melanoma subtype but also ethnicity influence the efficacy of anti-PD-1 ICI. Non- Caucasian had higher rates of skin and endocrine irAEs but low rates of pneumonitis than Caucasian. Following previous findings, current study conducted in Taiwan provides additionally important and valuable evidence showing high frequency of skin/endocrine irAE and low frequency of pneumonitis were associated with survivals in Asian melanoma undergoing PD-1 melanoma.

The results of previous studies regarding to irAEs and survival in melanoma patients undergoing therapy with anti-PD-1 ICI have been inconsistent. Okada et al. examined 15 melanoma patients undergoing nivolumab therapy and found that patients with irAEs were associated with better OS than patients without irAEs [9]. Indini et al. conducted a retrospective analysis of 173 patients with metastatic melanoma treated with anti-PD-1 antibodies and found that 59% of the patients experiencing irAEs showed improved PFS and OS, independent of other factors [16]. However, a large retrospective study analyzed the outcomes of 576 melanoma patients pooled from several studies treated with nivolumab [18] and no differences in PFS were found between patients with or without irAEs. Our cohort showed distinct results—that the prognostic value of irAE was severity dependent.

In terms of severity of irAEs, mechanistically as irAEs are considered to be the bystander effects of activation of T cells by ICIs, so patients who experience more severe irAEs should have higher T-cell activity and experience better outcomes than those who experience mild-to-moderate or no irAEs [24]. However, most of the previous studies on anti-PD-1 and anti-CTLA-4 antibodies rarely demonstrated the relationship between irAE severity and ICI efficacy. This variation in results could be attributed to the fact that patients experiencing severe irAEs tend to suffer from significant morbidity and sometimes mortality from the autoimmune reactions that compromise the benefit of ICIs [25]. In addition, severe toxicity is often associated with aggressive immunosuppression treatment, which may also influence the efficacy of ICIs [26]. Further, in the case of patients with severe irAEs, no ICIs were administered even after they completely recovered from the irAEs. Taken all together, these points could explain why the patients with severe irAEs had worse survival than patients without severe irAEs. One study in 858 older aged (≥65 years) melanoma patients treated with ipilimumab supported our findings as patients with non-severe irAE had improved OS compared to patients without irAE, and patients with severe irAE appeared to have the highest risk of death [27], although ipilimumab rather than anti-PD-1 was used in this study.

In current study, we found that different types of irAEs differentially predicted survival, as skin/vitiligo/endocrine irAEs were favorable irAEs. Sanlorenzo et al. found that patients with cutaneous irAEs had a significantly longer PFS than those without in a retrospective analysis of 83 metastatic cancer patients (including 66 melanoma patients) treated with pembrolizumab [28]. Yamazaki et al. followed 124 Japanese melanoma patients treated with nivolumab and reported that the occurrence of skin-related and endocrine-related irAEs had a significant impact on the PFS of the patients [29] although only the abstract is available currently. Moreover, Fujisawa et al. demonstrated that endocrine-related irAEs were associated with longer OS of melanoma patients treated with ipilimumab after nivolumab [30]. Other than melanoma, a significant correlation between endocrine irAEs and OS was observed (*p* = 0.019) in a pooling analysis of 12 randomized controlled trials of 3815 metastatic head and neck and lung cancer patients treated with ICIs [31]. All of above studies supported our finding that skin and endocrine irAEs represent favorable irAEs.

Vitiligo is a specific irAE in melanoma and is considered a predictive biomarker of the effectiveness of ICIs in advanced melanoma as T cells activated by ICIs may recognize the common antigens on tumor cells and host melanocytes. Therefore, the occurrence of vitiligo reflects that the T cells are activated and ready to kill the melanomas [32]. In a retrospective report, Indini analyzed various irAEs and found that vitiligo was associated with better OS than other irAEs although not a statistically significant level (*p* = 0.061) [16]. Only two patients experienced vitiligo in our cohort; therefore, the association between vitiligo and survival could not be undetermined.

Skin and endocrine irAEs in the current study were mild to moderate, so topical treatment and endocrine supplements were useful for managing the irAEs. Systemic steroids were not necessary for most of the patients who could continue ICI treatment. In contrast, pulmonary irAEs may be life threatening, so systemic steroids should be started as soon as pulmonary irAEs are suspected. The treatment course should be halted or terminated to improve the prognosis of patients with pulmonary irAEs. Fatigue is a nonspecific complaint resulting from ICI treatment, underlying malignancies or comorbidities. Therefore, patients with fatigue in our cohort may reflect disease progression rather than treatment-related AEs, which are difficult to differentiate in the initial presentation if patients have no other discomfort.

The association between irAEs and clinical outcomes was evident in not only metastatic melanoma but also resected melanoma with adjuvant therapy. In Keynote-054, pembrolizumab was found to improve recurrence-free survival (RFS) of stage III melanoma patients after complete resection [33]. The occurrence of an irAE was associated with longer RFS in the pembrolizumab arm, particularly endocrine AE [34]. Although patients appeared to have a low risk of recurrence or death after vitiligo onset in the pembrolizumab arm, statistical significance was not observed due to the limited number of cases (*n* = 24). In addition, the severe irAEs (grade 3–4) were not significantly associated with favorable RFS, but the patients who experienced severe irAEs seemed to be numerically worse than those had not experienced severe irAE in the pembrolizumab arm. These findings are compatible with our findings on advanced melanoma as the severity and types of irAE predict the survival outcomes.

As the concern of glucocorticoids/immunosuppressant may suppress the efficacy of ICIs [35], withholding ICIs rather than glucocorticoids unless great 3 irAE or pneumonitis [21]. The most frequent irAE in current study were skin and endocrine toxicities so topic glucocorticoids with or without oral antihistamine, and hormone replacement therapy were applied for those patients with skin and endocrine toxicities respectively. Only three patients experienced grade 3–5 irAE and two patients had pneumonitis, it is difficult to investigate the correlation and possible impact on survivals.

There are some limitations to the current retrospective study. Challenges of guarantee-time bias should be considered [36] because patients who experience irAEs are usually those who remain on ICI treatment for longer time periods and thus have a better prognosis than those who do not have irAEs. A retrospective study pooling melanoma patients from the randomized Checkmate 067 and Checkmate 069 trials suggested time in therapy is not the factor behind the relationship between irAE onset and ICI efficacy [37]. A Cox model with a time-varying covariate was used to avoid this bias for KEYNOTE-054, and the hazard of recurrence or death was lower in the pembrolizumab-treated patients after irAE onset (HR 0.37; 95% CI 0.24–0.57) than in those without or before irAE onset (HR 0.61; 95% CI 0.49–0.77) (*p* = 0.03) [34]. In current study, most skin irAE occurred in early period (< 30 days) of ICIs treatment indicating the early immune response of skin irAE may help clinician to predict the tumor response. In contrast, most endocrine irAE occurred after 3-month treatment so the prognostic value of endocrine irAE may be influenced by guarantee-time bias.

Small number of cases in current study limited the significance and some results should be interpreted cautiously. Only three patients experienced grade 3–5 irAE and two patients had pneumonitis so clinical significance of such irAE should not be confirmed using limited cases even statistical significance (*p* < 0.05, Figs. 2, 3). The current study did not analyze the impact of autoimmune diseases as preexisting autoimmunity may be associated with ICIs efficacy and irAE [38]. However, the previous studies showed ICIs lead to similar rates of irAEs in patients with coexisting autoimmune diseases compared with those without existing coexisting autoimmune diseases [39].

## Conclusion

In conclusion, melanoma patients undergoing anti-PD-1 ICIs who experienced mild-to-moderate irAE (grade 1–2) had favorable survival outcomes than those without irAE or severe irAE (grade 3–5). Patients with skin/vitiligo/endocrine irAEs had favorable survival. Therefore, the clinical outcomes of melanoma patients undergoing anti-PD-1 ICIs may be severity- and type-dependent, as per the current cohort.

## Supplementary information

**Additional file 1: Supplementary Figure S1.** Progression-free survival (PFS) and overall survival (OS) in melanoma patients receiving anti-PD-1 antibodies. The numbers below the charts correspond to patients at risk at each time point. **Supplementary Figure S2.** Progression-free survival (PFS) (A, C, E) and overall survival (OS) (B, D, F) in melanoma patients receiving anti-PD-1 antibodies, who had skin (A, B), vitiligo (C, D), and endocrine (E, F) irAEs. The patients experiencing skin/vitiligo (C, *p* = 0.092) and endocrine (E, *p* = 0.094) irAEs showed a trend of favorable PFS. The patients experiencing skin (*p* = 0.054), vitiligo (*p* = 0.047), and endocrine (*p* = 0.062) irAEs had a trend of favorable OS. The numbers below the charts correspond to patients at risk at each time point. irAEs, immune-related adverse events.

**Additional file 2: Supplementary Table 1.** Adverse events.

## Data Availability

The datasets generated AND analysed during the current study are not publicly available due to IRB regulation but are available from the corresponding author on reasonable request.

## Abbreviations

CGMH: Chang Gung Memorial Hospital
ECOG: Eastern Cooperative Oncology Group
HR: hazard radio
ICIs: immune checkpoint inhibitors
irAE: immune-related adverse event
OS: overall survival
ORR: overall response rate
PFS: progression-free survival
