# Supplementary material for: The association between immune-related adverse events and survival outcomes in Asian patients with advanced melanoma receiving anti-PD-1 antibodies
Source: BMC Cancer. 2020 Oct 21;20:1018. doi: 10.1186/s12885-020-07508-7 (PMC7579996; doi:10.1186/s12885-020-07508-7)
Supplement: Supplementary file 1 — Additional file 1: Supplementary Figure S1. Progression-free survival (PFS) and overall survival (OS) in melanoma patients receiving anti-PD-1 antibodies. The numbers below the charts correspond to patients at risk at each time point. Supplementary Figure S2. Progression-free survival (PFS) (A, C, E) and overall survival (OS) (B, D, F) in melanoma patients receiving anti-PD-1 antibodies, who had skin (A, B), vitiligo (C, D), and endocrine (E, F) irAEs. The patients experiencing skin/vitiligo (C, p = 0.092) and endocrine (E, p = 0.094) irAEs showed a trend of favorable PFS. The patients experiencing skin (p = 0.054), vitiligo (p = 0.047), and endocrine (p = 0.062) irAEs had a trend of favorable OS. The numbers below the charts correspond to patients at risk at each time point. irAEs, immune-related adverse events. [file 12885_2020_7508_MOESM1_ESM.pdf]

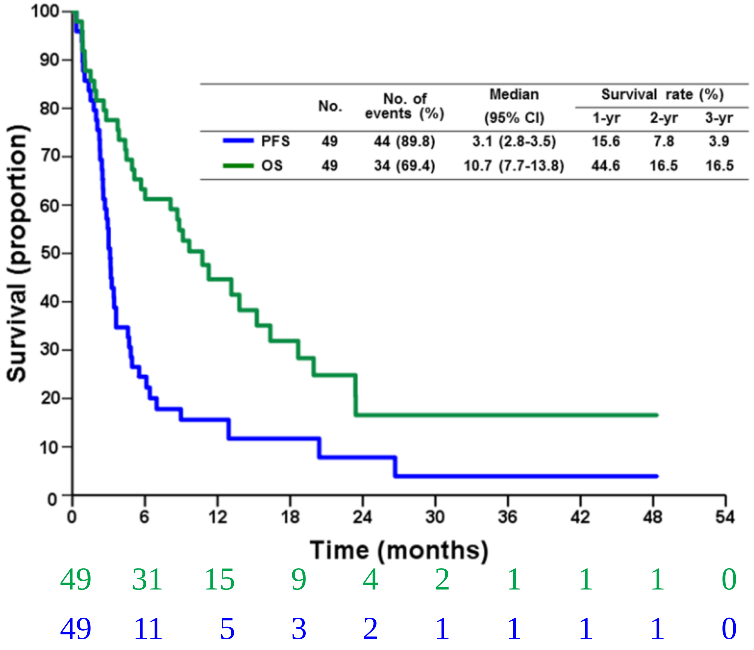

Supplementary Figure S1. Progression-free survival (PFS) and overall survival (OS) in melanoma patients receiving anti-PD-1 antibodies. The numbers below the charts correspond to patients at risk at each time point.

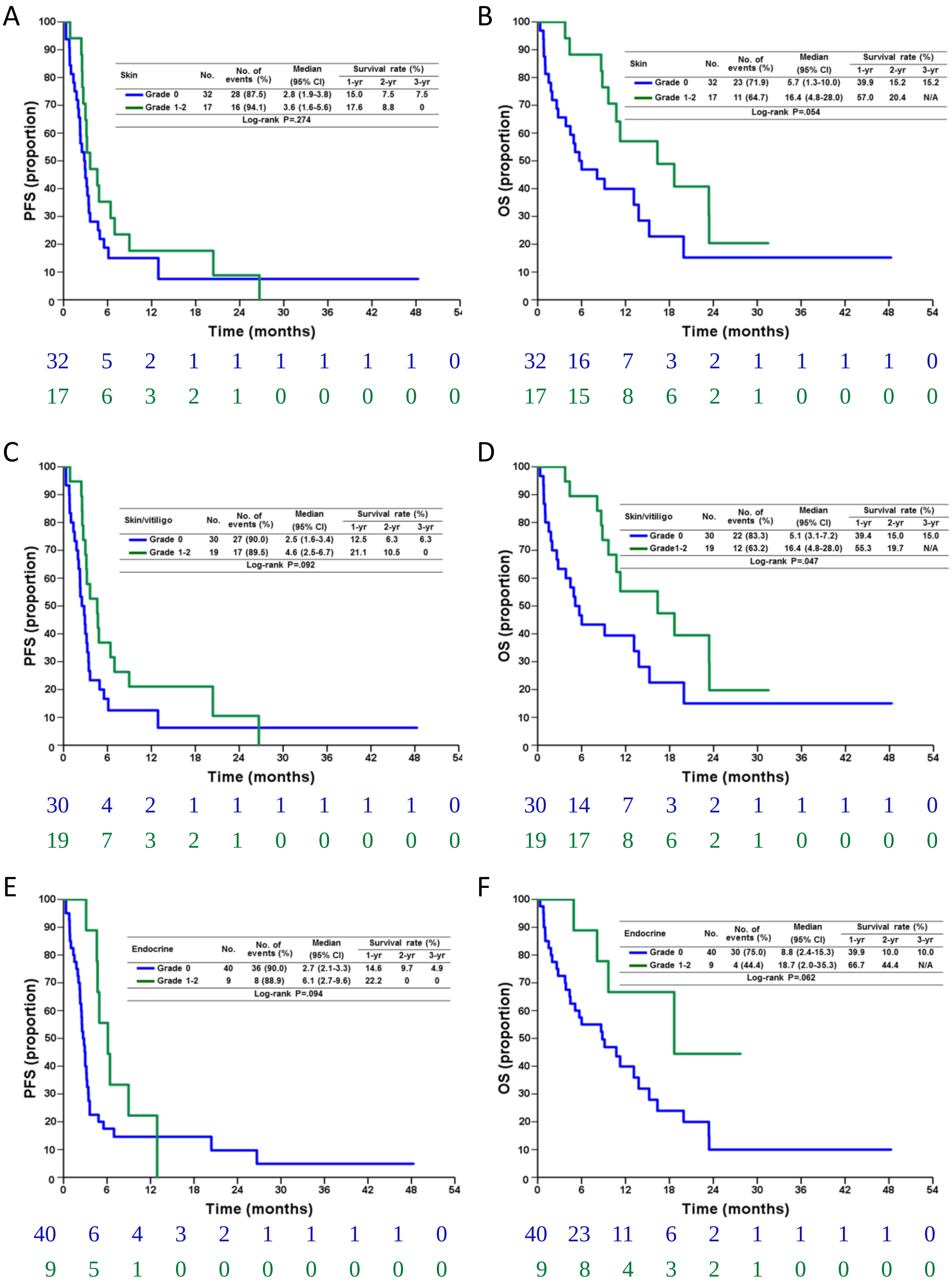

Supplementary Figure S2. Progression-free survival (PFS) (A, C, E) and overall survival (OS) (B, D, F) in melanoma patients receiving anti-PD-1 antibodies, who had skin (A, B), vitiligo (C, D), and endocrine (E, F) irAEs

The patients experiencing skin/vitiligo (C, p = 0.092) and endocrine (E, p = 0.094) irAEs showed a trend of favorable PFS. The patients experiencing skin (p = 0.054), vitiligo (p = 0.047), and endocrine (p = 0.062) irAEs had a trend of favorable OS The numbers below the charts correspond to patients at risk at each time point..

irAEs, immune-related adverse events
