# Supplementary material for: The association between immune-related adverse events and survival outcomes in Asian patients with advanced melanoma receiving anti-PD-1 antibodies
Source: BMC Cancer. 2020 Oct 21;20:1018. doi: 10.1186/s12885-020-07508-7 (PMC7579996; doi:10.1186/s12885-020-07508-7)
Supplement: Supplementary file 2 — Additional file 2: Supplementary Table 1. Adverse events. [file 12885_2020_7508_MOESM2_ESM.docx]

Supplementary Table 1. Adverse events

| Adverse events | Number. | % |
| --- | --- | --- |
| Skin |  |  |
| Grade 0 | 32 | 65.3 |
| Grade 1 | 12 | 24.5 |
| Grade 2 | 5 | 10.2 |
| Skin/vitiligo |  |  |
| Grade 0 | 30 | 61.2 |
| Grade 1 | 13 | 26.5 |
| Grade 2 | 6 | 12.2 |
| Mucositis |  |  |
| Grade 0 | 47 | 95.9 |
| Grade 1 | 1 | 2.0 |
| Grade 2 | 1 | 2.0 |
| Colitis |  |  |
| Grade 0 | 43 | 87.8 |
| Grade 1 | 4 | 8.2 |
| Grade 2 | 2 | 4.1 |
| Liver |  |  |
| Grade 0 | 47 | 95.9 |
| Grade 2 | 1 | 2.0 |
| Grade 3 | 1 | 2.0 |
| Lung |  |  |
| Grade 0 | 47 | 95.9 |
| Grade 3 | 1 | 2.0 |
| Grade 5 | 1 | 2.0 |
| Endocrine |  |  |
| Grade 0 | 40 | 81.6 |
| Grade 1 | 6 | 12.2 |
| Grade 2 | 3 | 6.1 |
| Fatigue |  |  |
| Grade 0 | 42 | 85.7 |
| Grade 1 | 6 | 12.2 |
| Grade 2 | 1 | 2.0 |
| Vitiligo |  |  |
| Grade 0 | 47 | 95.9 |
| Grade 1 | 1 | 2.0 |
| Grade 2 | 1 | 2.0 |
| Overall |  |  |
| Grade 0 | 19 | 38.8 |
| Grade 1 | 13 | 26.5 |
| Grade 2 | 14 | 28.6 |
| Grade 3 | 2 | 4.1 |
| Grade 4 | 0 | 0 |
| Grade 5 | 1 | 2.0 |
